# Early unexpected failure of a vitamin E-infused highly cross-linked polyethylene liner: A case report

**DOI:** 10.1097/MD.0000000000027454

**Published:** 2021-10-15

**Authors:** Kyeong Baek Kim, Sang-Min Lee, Nam Hoon Moon, Min Uk Do, Won Chul Shin

**Affiliations:** a Department of Orthopaedic Surgery, Pusan National University Yangsan Hospital, Yangsan, Republic of Korea; b Research Institute for Convergence of Biomedical Science and Technology, Pusan National University School of Medicine, Yangsan, Republic of Korea; c Department of Orthopaedic Surgery, Pusan National University Hospital, Busan, Republic of Korea.

**Keywords:** dual-mobility, liner, polyethylene, rim fracture, vitamin E-infused, wear

## Abstract

**Rationale:**

Total hip arthroplasty (THA) with a polyethylene (PE) liner is 1 of the most effective and successful treatment strategies for end-stage hip disorders. Vitamin E-infused highly cross-linked polyethylene is theoretically known to prevent failure due to oxidative degradation in the body, and is resistant to wear; therefore, successful long-term survival of THA is expected.

**Patient concerns:**

In June 2019, approximately 1.5 years after THA, the patient sat down and stood up without any specific issue; however, an unusual bullet sound occurred around the left hip joint. Since then, his discomfort persisted, and he was admitted to the emergency department.

**Diagnosis:**

Plain radiography and metal artifact reduction computed tomography performed in the emergency department revealed eccentric elevation of the prosthetic femoral head and suspected PE liner failure.

**Intervention:**

Revision surgery was performed for modular component exchange. To reduce the dislocation risk without performing cup exchange, conversion to dual-mobility articulation was performed.

**Outcomes:**

During the regular follow-up post-surgery, the patient could perform daily life activities without any discomfort, and dislocation was not observed. At 2 years postoperatively, no significant changes were observed in the radiographic images.

**Lessons:**

This case report presents an unexpected failure of THA due to superior rim fracture and excessive wear at the locking mechanism of the vitamin E-infused highly cross-linked polyethylene liner. This is an interesting case, as early PE liner failure occurred without strong labor intensity or trauma. A modular component exchange was performed with a dual-mobility bearing, and no issues were observed approximately 2 years after the reoperation. Therefore, third-generation highly cross-linked polyethylene liners can also cause early failure without a clear cause, and this case report highlights the necessity of considering several strategies for reoperation.

**Study Design:**

Case report.

## 1 Introduction

Total hip arthroplasty (THA) with a polyethylene (PE) liner is one of the most effective and successful treatment strategies for end-stage hip disorders.^[1,2]^ PE liner wear is an important factor that hinders the successful long-term survival of THA, and wear particulate debris causes osteolysis and implant loosening. The highly cross-linked polyethylene (HXLPE) liner, which undergoes electron-beam irradiation and remelting was introduced in 1998 to minimize or eliminate wear and decrease osteolysis and implant loosening associated with wear debris by non-cross-linked PE. However, free radicals generated by radiation during the cross-linking process may eventually cause mechanical failure of the PE liner through oxidative degradation in the body.^[3,4]^ There are methods to remove these free radicals, and among them, vitamin E-infused highly cross-linked polyethylene (VEPE) was made using vitamin E and its analogs and was available in 2010.^[5]^ VEPE is theoretically known to prevent failure due to oxidative degradation in the body, and is resistant to wear; therefore, successful long-term survival of THA is expected.

The authors report a case of rim failure with wear in a patient who underwent THA using VEPE. This highlights the importance of continuing research and development on PE liners, since failure may occur approximately 1.5 years after THA without any specific trauma, and unexpected early failure may occur even when using VEPE. In addition, we highlight the importance of preparing various strategies for reoperation, as well as the importance of early diagnosis when symptoms related to PE liner failure occur.

## 2 Case report

**Figure 1:**
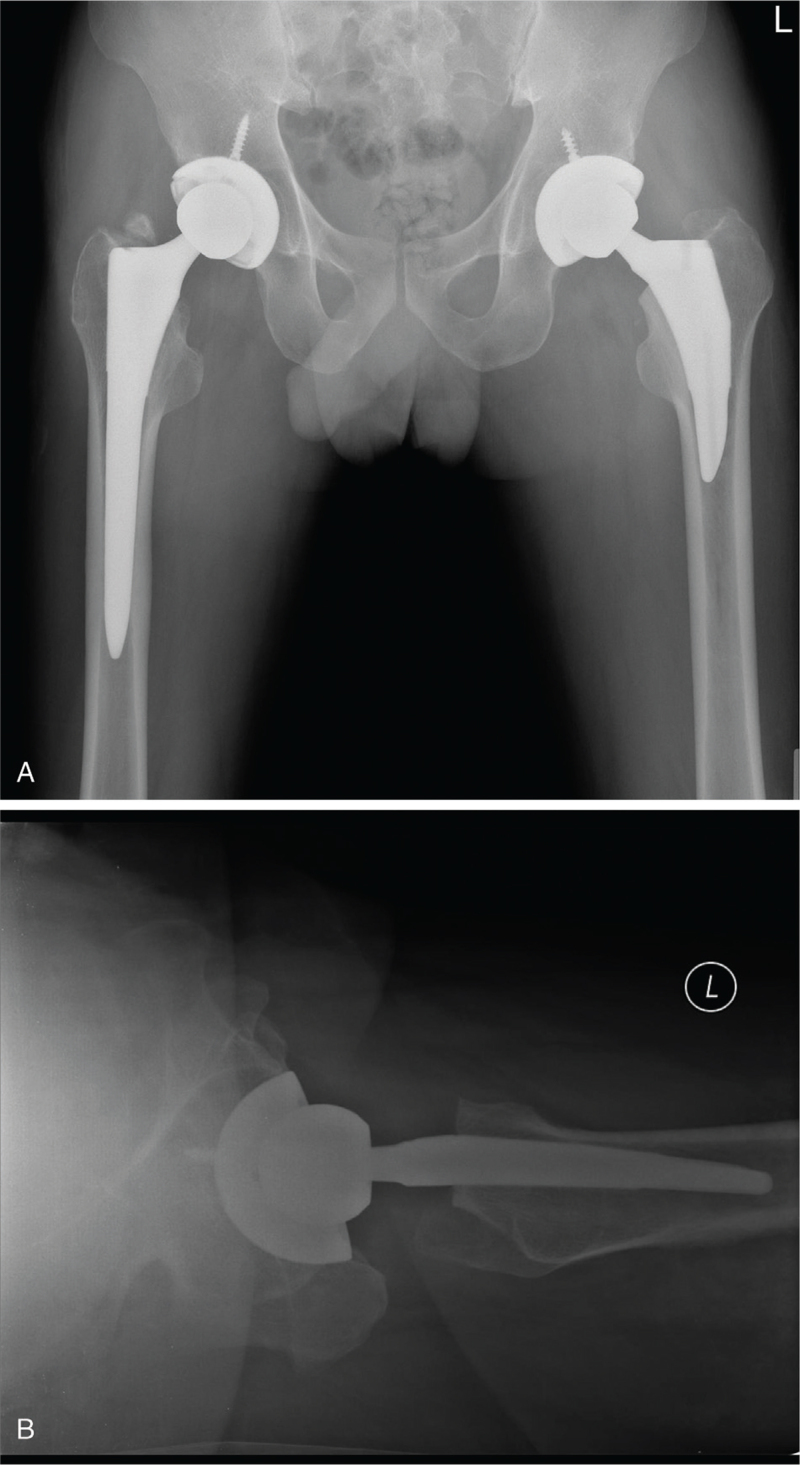
(A) Plain anteroposterior (AP) radiograph (B) cross-table lateral radiograph taken 6 weeks post-surgery showing acceptable position of the components.

In June 2017, a 50-year-old man who underwent THA at a local clinic for osteonecrosis of the right femoral head presented with left hip pain. Plain radiography performed at the time of admission revealed osteonecrosis of the left femoral head with femoral head collapse, which was determined to be at Ficat-Arlet stage III and Steinberg stage III. During preoperative evaluation, body mass index was 23.39 kg/m^2^. He had a normal bone mineral density (BMD) with no specific underlying disease, and primary THA was planned. In December 2017, cementless THA was performed using modified Gibson's posterolateral approach. The implants used were a 58-mm G7 cementless cup (Zimmer Biomet, Inc. Warsaw, IN), standard rim E1 polyethylene liner (Zimmer Biomet), 36-mm, -3.0 mm neck length ceramic head (Biolox Delta), and standard offset size 13 Microplasty stem (Zimmer Biomet). Partial weight-bearing ambulation using crutches was started on the first day post-surgery, and full weight-bearing was allowed 6 weeks after surgery. On plain radiographs taken 6 weeks post-surgery, the proper position of the implant was confirmed with an inclination angle of approximately 46° and an anteversion angle of approximately 18° (Fig. 1). The patient was a general laborer, who was able to work without any restrictions in terms of special activities. After 3 months, 6 months, and 1 year postoperatively, regular follow-up was performed every year.

**Figure 2:**
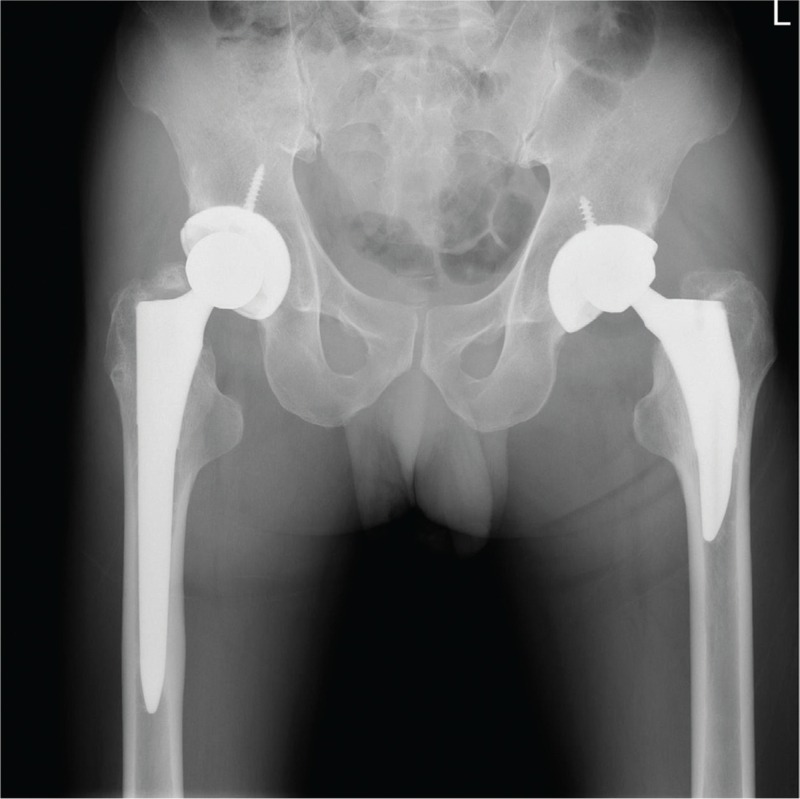
Plain AP radiograph taken 1.5 years post-surgery showing eccentric elevation of prosthetic femoral head. AP = anteroposterior.

**Figure 3:**
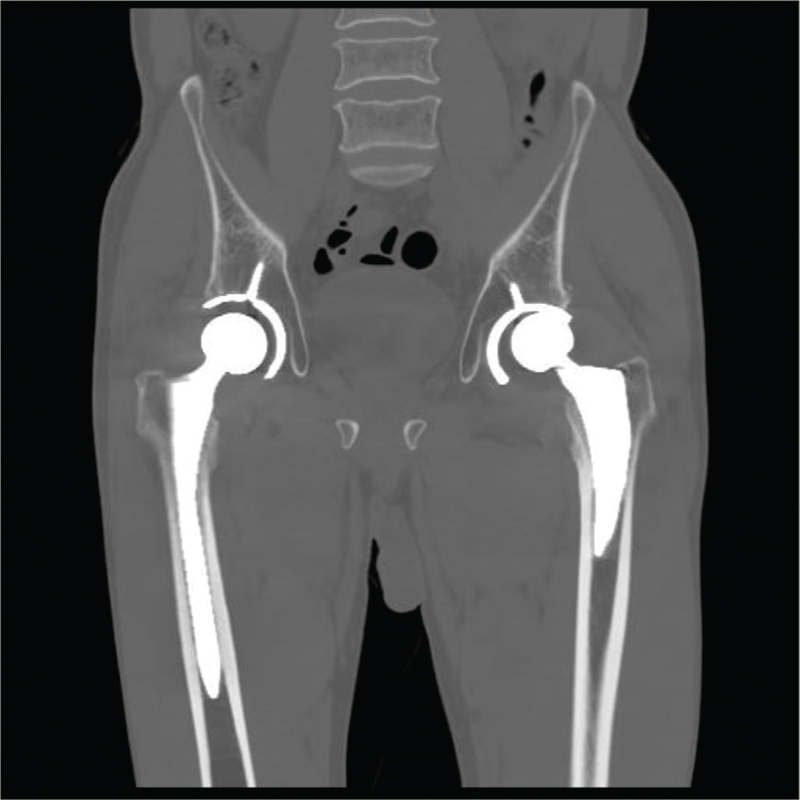
Metal artifact reduction computed tomography (MAR-CT) showing suspected polyethylene liner failure. MAR-CT = metal artifact reduction computed tomography.

**Figure 4:**
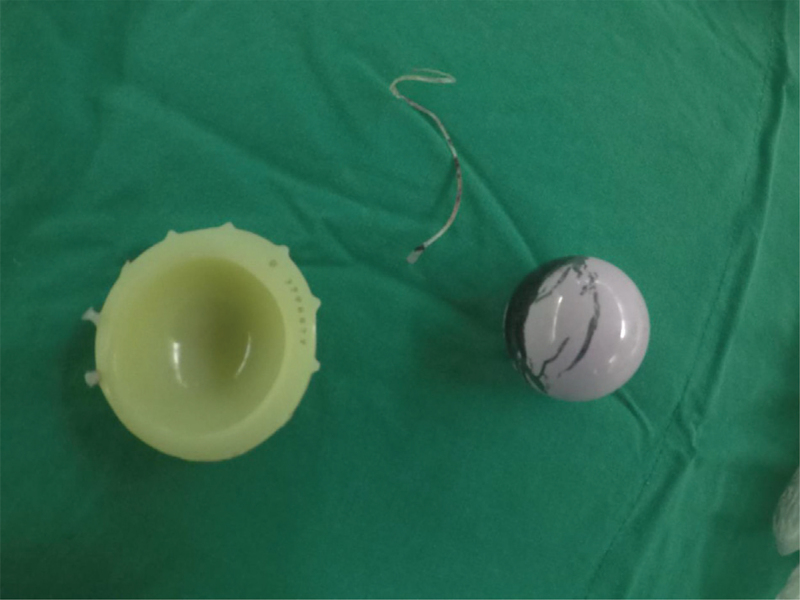
Photographs of superior rim fracture and excessive wear of the polyethylene liner. Striped markings on the prosthetic femoral head by metal transfer after removing the liner.

In June 2019, approximately 1.5 years after THA, the patient sat down and stood up casually; however, an unusual bullet sound occurred around the left hip joint. Since then, his discomfort persisted, and he was admitted to the emergency department. Plain radiography and metal artifact reduction computed tomography performed in the emergency department revealed eccentric elevation of the prosthetic femoral head and suspected PE liner failure (Figs. 2 and 3). Revision surgery was performed for modular component exchange, as well as for potential cup revision. Intraoperative findings showed that the PE liner was separated from the acetabular shell and moved down the joint cavity. Furthermore, superior rim fracture of the liner, including at the locking groove, and excessive PE wear in the same area, were observed. Striped markings were observed on the prosthetic femoral head by metal transfer, and marking due to friction was observed at the metal shell after removing the liner (Fig. 4). However, the shell was relatively intact, and stable fixation was confirmed. No gross infection was found in the operative field, and a frozen section biopsy revealed normal findings. If the cause of PE liner failure could not be clearly identified, and if the VEPE liner was re-used during revision, the high risk of dislocation after the modular component exchange and reoccurrence of PE liner failure were considered. The G7 cup used as the primary THA was converted to a 46-mm metal insert and dual-mobility PE (E1) head (Zimmer Biomet), since this system is capable of dual-mobility articulation. As it was confirmed that there was no damage at the stem taper, a modular component exchange was performed using a 28-mm, +0 mm neck length ceramic head (Biolox Delta).

**Figure 5:**
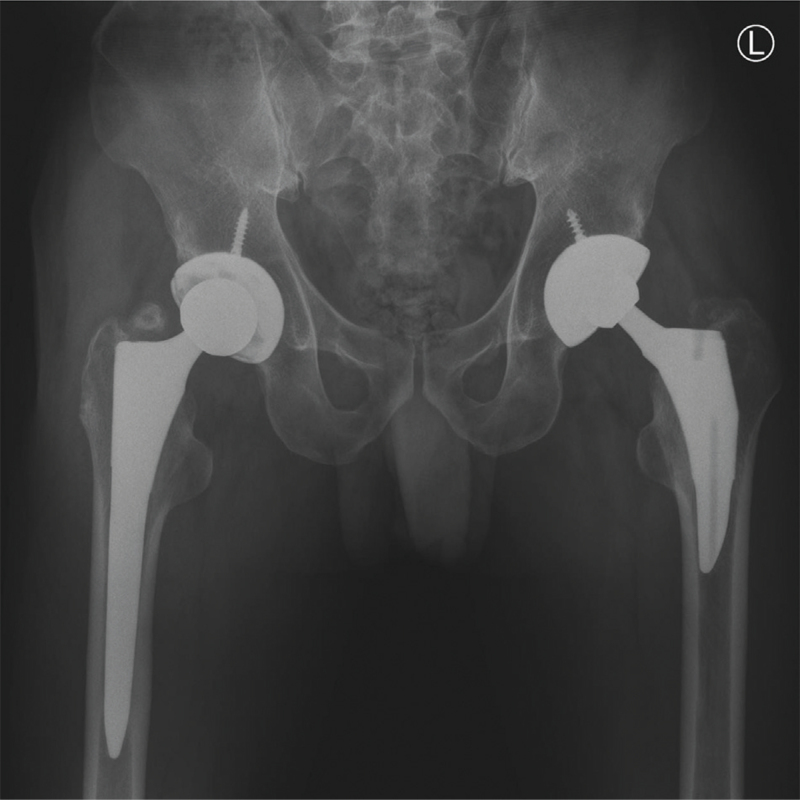
AP radiographs of the pelvis taken 2 years post-surgery following revision of hip components. AP = anteroposterior.

During the regular follow-up post-surgery, the patient could perform daily life activities without any discomfort, and dislocation was not observed. At 2 years postoperatively, no significant changes were observed in the radiographic images (Fig. 5).

## 3 Discussion

Aseptic loosening caused by wear particles of ultra-high molecular weight polyethylene (UHMWPE) has been reported as a primary cause of THA failure.^[6]^ Since then, the development of bearing surfaces for the long-term survival of THA has continued. When UHMWPE is irradiated beyond the normal sterilization dose (25 to 40 kGy), highly cross-linked UHMWPE is formed, which has demonstrated dramatically improved wear characteristics over UHMWPE. Residual free radicals inevitably generated during the cross-linking process cause PE failure through oxidative degradation in the human body.^[3,4]^ Remelting (above the melting temperature of the polymer) eliminates free radicals almost entirely, but compromises the fatigue strength of the material and can absorb lipids, which can also lead to oxidation.^[7]^ Annealing, or heating to a temperature just below the melting point of the polymer reduces the number of free radicals to a lesser extent than that of melting, but the strength of the material is better preserved.^[8]^ In other words, intrinsic limitations remain in terms of the ideal PE properties for both the remelting and annealing processes. Recently, VEPE was introduced as a potential solution to this problem.^[5]^ Free radical scavengers are added to PE during processing, and vitamin E adequately quenches free radicals that remain after irradiation without a post-irradiation heating step. Therefore, vitamin E reduces the risk of oxidation without sacrificing the strength or wear properties of the polymer.

Several in vitro studies have shown that VEPE has better wear resistance than conventional HXLPE.^[9,10]^ Halma et al reported that VEPE was approximately 5–6 times more resistant than UHMWPE when comparing bonds resistant to edge loads using a 28-mm prosthetic femoral head. In an in vivo study, when comparing the wear degree of VEPE and mechanically annealed UHMWPE (ModXLPE), both PEs showed low wear rates at the 2-year follow-up. However, while the degree of wear was similar, VEPE showed a lower wear rate at the 5-year follow-up.^[11]^ In 1 study comparing the generation of wear particles between VEPE and conventional PE in total knee arthroplasty, synovial fluid analysis was performed on an average of 3.4 years postoperatively. The number of wear particles in the VEPE group was approximately 1.5 times that of conventional PE. Furthermore, the particle size was approximately 3 times smaller.^[12]^ In a recent randomized controlled trial study involving 199 patients, VEPE had a higher Harris hip score than HXLPE, and a femoral head penetration rate of 0.046 mm/yr, which was lower than that of HXLPE (0.056 mm/yr).^[13]^ However, there are few in vivo studies; therefore, it will be necessary to observe continuous results in the future.

HXLPE failure and rim fractures have been reported several times due to oxidative degradation of residual free radicals, but only 2 cases of VEPE liner rim fractures have been reported to date.^[14,15]^ Liner thickness, design defaults, trauma history, and improper implant position were presumed to be possible causes. However, no clear cause was found in either case, and further studies are warranted. In this case report, a rim fracture occurred in the same area, along with wear of the VEPE. This differs from the previous report, in which fracture was not accompanied by wear. In this case, a 36-mm ceramic head was used in a 58-mm cementless hemispherical cup. Compared to the metal prosthetic head used in other cases, this is a bearing combination known to have a relatively superior long-term survival.^[16]^ In addition, it is known that the risk of PE rim fractures increases when peripheral liner thickness is insufficient. Waewsawangwong et al reported that the HXPE thickness should be at least 6 mm to reduce the occurrence of liner failure.^[17]^ Ast et al proposed a thickness of 7 mm in the weight-bearing area and 4.8 mm in the rim.^[18]^ In this case, the minimum liner thickness was 7.7 mm at the apex, 7.3 mm at 45° angle, and 5.8 mm at the rim. Compared to the thicknesses of other cases reporting PE liner failure, a relatively sufficient thickness was confirmed. In addition, unlike other previous reports, liner failure occurred when the implant position was appropriate, and no specific trauma or infection had occurred. The authors could not identify the exact cause, and concluded that a cause different from that previously reported could have occurred. Furthermore, it cannot be concluded that VEPE is a PE with ideal wear and mechanical properties.

In this case, a modular component exchange using dual-mobility articulation was performed during the revision. This is because the cause of the failure of VEPE was not clear, so there was concern of rim fracture recurrence with only VEPE exchange. Furthermore, the risk of postoperative dislocation is approximately 3 times higher with revision THA than with primary THA. In particular, the dislocation rate may increase further in the case of isolated liner exchange. The G7 cup used in our patient was a multiple optional cup that could use both PE, ceramic, and dual articulation. On the radiographic images evaluated before revision, the implant position was determined to be appropriate, and the cup fixation was stable. To reduce the dislocation risk without performing cup exchange, conversion to dual-mobility articulation was performed. In particular, because the patient experienced discomfort and the diagnosis was not delayed, damage to the metal shell was minimized; thus, a relatively simple revision could obtain good results.

## 4 Summary

This is the first case of failure accompanied by wear of the VEPE liner, which is known to be very resistant to wear, at an early stage of 1.5 years after surgery. There was no history of trauma; the ceramic head-VEPE liner bearing combination was known to have excellent long-term survival, and the implant position and liner thickness were sufficiently secured compared to other cases. The patient visited the emergency department 2 days after the onset of symptoms, was quickly diagnosed with liner failure, and underwent revision surgery by preparing various strategies before surgery. Although the cause of VEPE wear was not identified, this report demonstrated that VEPE can also cause wear and lead to unexpected early failure, and further studies and case reports are needed to identify the cause of VEPE wear and the minimum thickness of VEPE required. Furthermore, this case report highlights the importance of considering multiple surgical options, such as dual-mobility articulation, when symptoms related to PE liner failure occur, and when reoperation is performed when the cause of VEPE failure is unclear. This can reduce the likelihood of dislocation and the recurrence of symptoms. This is an implication of this case report.

## Author contributions

**Writing – original draft:** Kyeong Baek Kim.

**Writing – review & editing:** Sang-Min Lee, Nam Hoon Moon, Min Uk Do, Won Chul Shin.
